# Dual-Cell Patch-Clamp Recording Revealed a Mechanism for a Ribbon Synapse to Process Both Digital and Analog Inputs and Outputs

**DOI:** 10.3389/fncel.2021.722533

**Published:** 2021-10-14

**Authors:** Ji-Jie Pang, Fan Gao, Samuel M. Wu

**Affiliations:** Department of Ophthalmology, Baylor College of Medicine, Houston, TX, United States

**Keywords:** retina, photoreceptor, bipolar cell, glutamate synapse, dual-cell patch-clamp, light response, ganglion cell

## Abstract

A chemical synapse is either an action potential (AP) synapse or a graded potential (GP) synapse but not both. This study investigated how signals passed the glutamatergic synapse between the rod photoreceptor and its postsynaptic hyperpolarizing bipolar cells (HBCs) and light responses of retinal neurons with dual-cell and single-cell patch-clamp recording techniques. The results showed that scotopic lights evoked GPs in rods, whose depolarizing Phase 3 associated with the light offset also evoked APs of a duration of 241.8 ms and a slope of 4.5 mV/ms. The depolarization speed of Phase 3 (Speed) was 0.0001–0.0111 mV/ms and 0.103–0.469 mV/ms for rods and cones, respectively. On pairs of recorded rods and HBCs, only the depolarizing limbs of square waves applied to rods evoked clear currents in HBCs which reversed at −6.1 mV, indicating cation currents. We further used stimuli that simulated the rod light response to stimulate rods and recorded the rod-evoked excitatory current (rdEPSC) in HBCs. The normalized amplitude (R/R_max_), delay, and rising slope of rdEPSCs were differentially exponentially correlated with the Speed (all *p* < 0.001). For the Speed < 0.1 mV/ms, R/R_max_ grew while the delay and duration reduced slowly; for the Speed between 0.1 and 0.4 mV/ms, R/R_max_ grew fast while the delay and duration dramatically decreased; for the Speed > 0.4 mV/ms, R/R_max_ reached the plateau, while the delay and duration approached the minimum, resembling digital signals. The rdEPSC peak was left-shifted and much faster than currents in rods. The scotopic-light-offset-associated major and minor cation currents in retinal ganglion cells (RGCs), the gigantic excitatory transient currents (GTECs) in HBCs, and APs and Phase 3 in rods showed comparable light-intensity-related locations. The data demonstrate that the rod-HBC synapse is a perfect synapse that can differentially decode and code analog and digital signals to process enormously varied rod and coupled-cone inputs.

## Highlights

- It is unclear how a chemical synapse deals with both graded and spiking drives.
- It is uncertain how rod pathways mediate excitatory transient OFF visual signals.
- Hyperpolarizing bipolar cells (HBCs) were found to encode rod depolarization speed.
- Rod-HBC ribbon synapses outputted analog and digital-like signals.
- Rod and coupled cone inputs each fall in both the analog and digital zones.

## Introduction

Chemical synapses have long been classified as either action potential synapses or graded potential synapses (Wilson, 2004; Sterling and Matthews, 2005; Heidelberger, 2007). At action potential synapses, presynaptic neurons typically encode neuronal information into the frequency of action potentials, which pass the long axons to elicit action potentials in postsynaptic neurons. In such conventional action potential synapses, graded potentials are generated in presynaptic neurons but do not reach postsynaptic neurons. In contrast, in graded potential synapses, such as ribbon synapses, presynaptic neurons typically encode neuronal information into both amplitude and frequency (Baden et al., 2013; Grabner et al., 2016) of graded potentials, which, by modifying neurotransmitter release at the axonal terminals, could reach postsynaptic neurons and be spatially and temporally integrated there. Action potential and graded potential synapses use different mechanisms for neurotransmitter release. At action potential synapses, an action potential triggers a brief burst of exocytosis of neurotransmitters (Bean, 2007; Plomp et al., 2018), while synaptic ribbons in photoreceptors maintain glutamate release in darkness, which is graded with respect to Ca^2+^ influx induced by the presynaptic membrane depolarization (Heidelberger, 2007; Sanes and Zipursky, 2010) and/or Ca^2+^-independent (Chen et al., 2014). Therefore, as two distinct categories of neuronal structure, synaptic signals appear to be digital in the action potential synapse but analog at the graded potential synapse. However, in the central nervous system, some neurons using ribbon synapses have been found to generate both graded potentials and action potentials, such as photoreceptors (Fain et al., 1980; Kawai et al., 2001, 2005) and cone bipolar cells (Protti et al., 2000; Saszik and DeVries, 2012). In the explanted frog sacculus, hair cells can generate both spikes and fast membrane oscillations, mediating the periodic afferent activity (Rutherford and Roberts, 2009). While these studies point out that digital and non-digital inputs may both influence the synaptic output, it remains a fundamental question of how a chemical synapse encodes both spikes and the graded analog input into the analog or digital output signal (Baden et al., 2013).

Rods represent the majority of retinal photoreceptors, and they initiate scotopic vision. Rods primarily respond to light ON signals, which raises an essential question whether or how the excitatory scotopic OFF-center response observed in retinal ganglion cells (RGCs) and amacrine cells (Hensley et al., 1993; Volgyi et al., 2004; Pang et al., 2010b, 2016) could be generated in rod pathways (Wassle et al., 2009; Dowling, 2012; Masland, 2012) in the vertebrate retina. Rods make sign-preserving electrical synapses with cones and ribbon synapses with retinal bipolar cells (Rao-Mirotznik et al., 1995; Sterling and Matthews, 2005), the sign-preserving chemical synapse in the hyperpolarizing bipolar cell (BC) (HBC, OFF BC), and the sign-inverting one in the depolarizing BC (DBC). In photoreceptors, light closes cGMP-gated cation channels and hyperpolarizes the membrane, which reduces glutamate release to activate mGluR6 in DBCs and inhibit iGluRs in HBCs (Cadetti et al., 2005; Li et al., 2010). The connection between the rod—rod BC—AII amacrine—cone BC forms the so-called primary rod pathway, which is unique for mammals and critical for the excitatory ON signaling. The rod-cone coupling and the rod-HBC route are known as the secondary (Wu and Yang, 1988; Yang and Wu, 1989; Bloomfield and Dacheux, 2001) and tertiary (Soucy et al., 1998; Hack et al., 1999; Tsukamoto et al., 2001; Li et al., 2004; Pang et al., 2004) rod pathway, respectively, which are shared among vertebrates (Wassle et al., 2009; Dowling, 2012; Masland, 2012). Rod pathways had long been thought to be pure ON pathways until the rod ribbon synapse was found in HBCs (Soucy et al., 1998; Hack et al., 1999; Tsukamoto et al., 2001; Li et al., 2004; Pang et al., 2004). Since then, rod-HBC synapses have been believed to mediate OFF response in RGCs. Consistent with this idea, the vesicle fusion and turnover in mammalian rod ribbons were found to be fast, and rod synapses are further postulated to mediate rapid rod signaling (Rabl et al., 2006; Li et al., 2010). However, although rods and cones can both quickly hyperpolarize at the light onset to accurately signal light onset, rod repolarization often falls behind light offset (Toyoda et al., 1970; Xu et al., 2005; Pang et al., 2012b; Fortenbach et al., 2015), and cones presumably provide no signals to rods in the scotopic range. Thus, whether and how the well-accepted second and tertiary rod pathways mediate the excitatory scotopic OFF visual signal (Hensley et al., 1993; Volgyi et al., 2004; Pang et al., 2010b; Pan et al., 2016) remains an essential gap in visual neuroscience yet to be filled.

We hypothesize that the rod-HBC synapse mediates the scotopic OFF response and uses both analog and digital inputs and outputs. We studied the signal transmission at the rod-HBC synapse with dual-cell patch-clamping techniques and rod action potentials and light responses of rods, BCs, and RGCs with single-cell patch-clamp techniques in dark-adapted salamander retinas, given the similarity of the rod-HBC synapse (Pang et al., 2004) and rod-cone coupling (Wu and Yang, 1988; Yang and Wu, 1989; Gao et al., 2013) with the tertiary and the secondary pathways of mammals.

## Results

To understand the signal transmission of the rod-HBC synapse, we first studied the synaptic input by analyzing the waveform and time course of light responses of rods and cones. Based on the values of the rod and cone signals, we then designed electric stimuli that mimicked the light responses of photoreceptors to stimulate rods and quantified the input-output relationship of the rod-HBC synapse. The light response of rods, HBCs, and RGCs were recorded and further analyzed to determine the role of the rod-HBC synapse and the rod action potential in scotopic OFF signaling.

### Dark-Adapted Rods Responded to Light With Graded and Action Potentials

**FIGURE 1:**
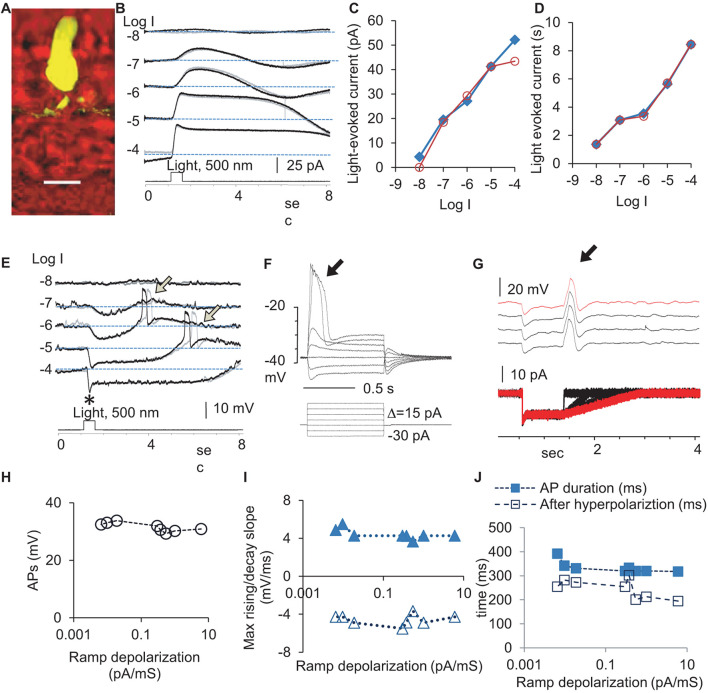
Light- and electric stimulus-evoked graded and action potentials (APs) in dark-adapted rods. Rods were recorded under whole-cell voltage- (**B**, Vh = –40 mV) and current-clamp modes **(E)**. **(A)** A recorded rod was filled with Lucifer yellow, and the scale bar is 20 μm. **(B)** Rods respond to 0.5 s light steps of various light intensities (in log unit, log I) generally with the outward current/hyperpolarization, which shows a progressively larger amplitude **(C)** and longer duration **(D)** with the increase of light intensity. **(E)** Light also evokes APs (arrow) after the light offset. The black and gray traces illustrate two trials recorded under the same light intensity **(B,E)**. The delay of APs evoked by the light offset shows some variation. Asterisk: a brief “nose” appears upon the photopic light onset. **(F–J)** The leading edge of the supra-threshold square-wave depolarization **(F)** and the trailing edge of the hyperpolarization specially designed to simulate rod light response **(G)** both evoked action potentials (arrow) of a stable amplitude **(H)**, duration **(J)**, and the rising and decay slopes **(I)**, consistent with the “all-or-none” principle of APs. **(F)** The voltage-dependent activation of APs in a rod with a resting potential of –46 mV and the threshold of the AP at ∼–36 mV. **(G)** The depolarizing ramp of various slopes at the end of the hyperpolarizing step of –15 pA, which mimics the rod Phase 3, elicits a single AP in rods (subthreshold stimuli are not presented). Vh-holding potential. The intensity of unattenuated [0 in log unit (log I)] 500 nm light from a halogen light source was 4.4 × 10^5^ photons.μm^–2^.s^–1^.

We first quantified the physiological signals feeding to the rod-HBC synapse by examining light-evoked currents (Figures 1B–D) at the membrane potential level (−40 mV) and light-evoked potentials (Figure 1E) at different light intensities at the holding current (Ih = 0) in rods (Figure 1). The light-evoked responses were primary graded responses, characterized by a progressively larger amplitude (Figure 1C) and longer duration (Figure 1D) upon increasing the light intensity. Light also evoked action potentials APs of a stable amplitude and duration after the light offset (Figure 1E). The leading edge of a depolarization step (Figure 1F) and a trailing edge of a hyperpolarization step (Figure 1G) both evoked APs. In Figure 1G, the stimulus was designed to have variable depolarizing slopes at the end to mimic the light response of rods at different light intensities. APs were evoked at the beginning of the ramp depolarization. The results indicate that both analog and digital inputs are present for the rod-HBC synapses.

In the dark-adapted rods, APs showed a duration of 241.8 ± 6.4 ms (*n* = 12), rising speed of 4.43 ± 0.19 mV/ms, decaying speed of 4.58 ± 0.2 mV/ms, and amplitude of 31.53 ± 0.54 mV. The delay time was *shorter* at lower light intensities. The electric-stimulus-evoked APs in rods showed voltage-dependent activation with a threshold of ∼−36 mV (Figure 1F), and the amplitude, duration, and slope of APs were stable (Figures 1F–J), consistent with the “all-or-none” property. About 67% rods did not show light-evoked APs, which was probably due to the presence of a stronger electrical coupling.

### The Comparison of the Kinetics and Delay of the Depolarization at the Light Offset With That of the Hyperpolarization at Light Onset in Photoreceptors

**FIGURE 2:**
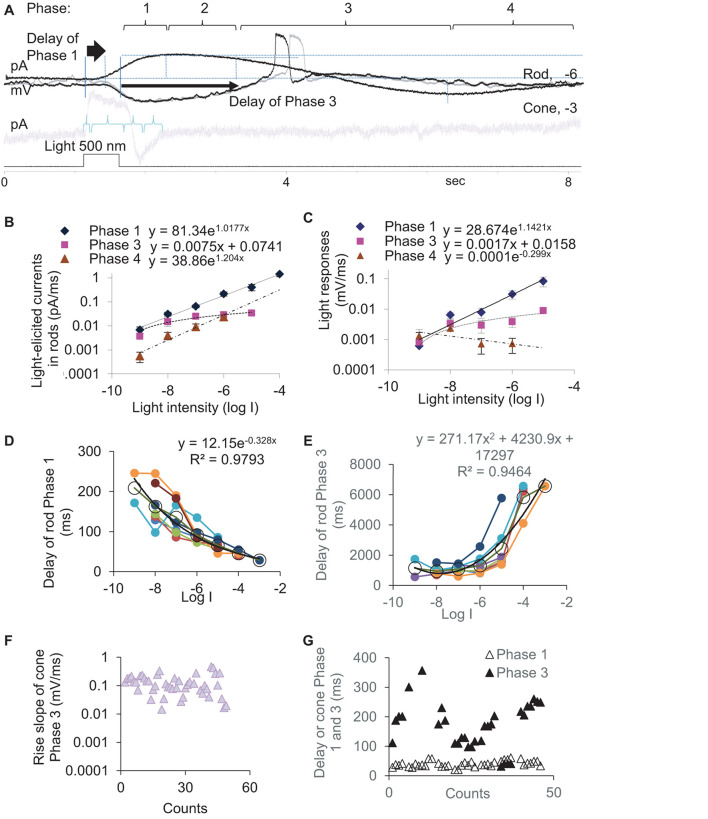
Kinetics and latency of the light response of photoreceptors. **(A)** The light-evoked current of rods and cones are described by Phase 1–4. Rods **(A–E)** or cones **(F,G)** were recorded under whole-cell voltage- (**A,B,D,E,G**, Vh = –40 mV) and current-clamp modes **(A,C,F)**. **(B,C)** The slope of light-evoked graded currents **(B)** and potentials **(C)** was exponentially correlated with light intensity in Phase 1 (diamond, *p* < 0.0001 and *p* = 0.001, respectively) and Phase 4 (triangle, *p* = 0.021 and *p* = 0.416, respectively) and linearly correlated with light intensity in Phase 3 (square, *p* = 0.004 and *p* = 0.041, respectively). **(D,E)** In rods, the delay time of Phase 1 **(D)** and Phase 3 **(E)** are exponentially *negatively* and *positively* correlated with the light intensity (log I), respectively (both *p* < 0.0001). Black circles and lines display the averaged delay (mean, SEM) and the fitting curve of data from individual rods (color dots and lines), respectively. **(F,G)** Light-evoked potentials **(F)** and currents **(G)** at –3 and –4 log I in cones. **(F)** Phase 3 slope of cones is ∼10 times faster than that of rods **(C)**. **(G)** The cone Phase 3 delays ∼10 times shorter than that of the rod Phase 3 **(E)**, while the delay of Phase 1 under photopic light is similar for rods **(D)** and cones. Vh-holding potential. The intensity of unattenuated [0 in log unit (log I)] 500 nm light from a halogen light source was 4.4 × 10^5^ photons.μm^–2^.s^–1^.

To better understand the synaptic input in the rod-HBC synapse, we divided the light response of photoreceptors into four phases per the polarity, polarization speed, and delay (Figure 2). Phase 1 included the transient outward current/hyperpolarization evoked by the light onset, which began from 10% of the peak and ended at 100% of the peak, and the delay was measured from light onset to 10% of the peak. Phase 2 was the portion with the sustained outward current/hyperpolarization after Phase 1, which began from 100% of the peak to where the sustained component reduced by 10%. Phase 3 began from 90% of the sustained outward current/hyperpolarization peak and ended at the turning point, whose delay was measured from light offset to 90% of the peak of the sustained current. Phase 4 is the small slow outward current/repolarization after Phase 3, which began from the turning point and ended at the resting level. The rising slope for Phase 1 and Phase 4 and the decaying speed for Phase 3 were measured between 30 and 70% of related limbs, where the slope was usually the steepest. The response of rods to brighter light often showed a “nose” between Phase 1 and Phase 2 (Figure 1E, asterisk), a fast brief hyperpolarization at the light onset, whose duration measured 31.97 ± 4.28 ms (*n* = 7) at the base.

In rods, the slope of light-evoked graded currents (Figure 2B) and potentials (Figure 2C) was exponentially correlated with light intensity in Phase 1 (*p* < 0.0001 and *p* = 0.001, respectively) and Phase 4 (*p* = 0.021 and *p* = 0.416, respectively) and linearly positively correlated with light intensity in Phase 3 (*p* = 0.004 and *p* = 0.041, respectively). The depolarization speed in Phase 3 ranged between 0.0001 and 0.0111 mV/ms at −9 to −5 log I (*n* = 27), and the steepest slopes were often observed under scotopic lights between −5 and −6 log I in our experimental conditions, which also evoked APs (Figure 1E). The cone light threshold is close to −4 (Yang and Wu, 1996). At −3 and −4 log I, the depolarizing slope of the cone Phase 3 was 0.103–0.469 mV/ms and averaged 136.9 ± 41.9 μV/ms (*n* = 46) (Figure 2F). Rod Phase 2 was sustained, and Phase 4 was shallow and slow, which were not further studied.

We also measured the delay time of the light response (Figures 2D,E) in dark-adapted rods (*n* = 8) at different light intensities and cones (*n* = 38) (Figure 2G) at −3 and/or −4. The delay time of the rod Phase 1 and Phase 3 are exponentially *negatively* and *positively* correlated with the light intensity (log I), respectively (both *p* < 0.0001). Phase 3 of rods delayed 944.7 ± 110.9 ms, 1085.9 ± 105.0 ms, 1308.0 ± 245.7 ms, and 2436.6 ± 595.9 ms at −8, −7, −6, and −5 log I, respectively. The best temporal resolution for rod-driven OFF responses (rOFF) was calculated to be 0.945 s (1.058 Hz) (Figure 2E). Phase 1 of rods delayed 29–209 ms at −8 to −3 log I (Figure 2D) and averaged 56 ± 4.81 ms (range 29–80 ms) at −3, −4 and −5 log I. Phase 3 of cones delayed 158.5 ± 13.9 ms at −4 and −3 log I (range 41–357 ms), which was significantly shorter than that of rods (*p* < 0.0001). Phase 1 of cones delayed 40.22 ± 1.60 ms (range 21–61 ms) at −3 and −4 log I and was not statistically different from that of rods at −4 log I (*p* = 0.267).

Because the light threshold of rods and cones is ∼3.5 log unit apart and the rod Phase 3 delays much longer in rods than that of cones, the data demonstrate that the rod- and cone-driven response to the light offset in postsynaptic neurons are temporally separated and cannot be integrated or mixed as their response to light onset can be. Also, the depolarization speed of light-evoked analog signals of rods, analog signals of cones, and digital signals/APs of rods were separated into three levels, which were < 0.01 mV/ms, 0.1–0.4 mV/ms, and ∼4.5 mV/ms, respectively.

### How Does the Depolarization Speed of the Rod Input at the Rod-Hyperpolarizing Bipolar Cell Synapse Determine the Synaptic Output?

**FIGURE 3:**
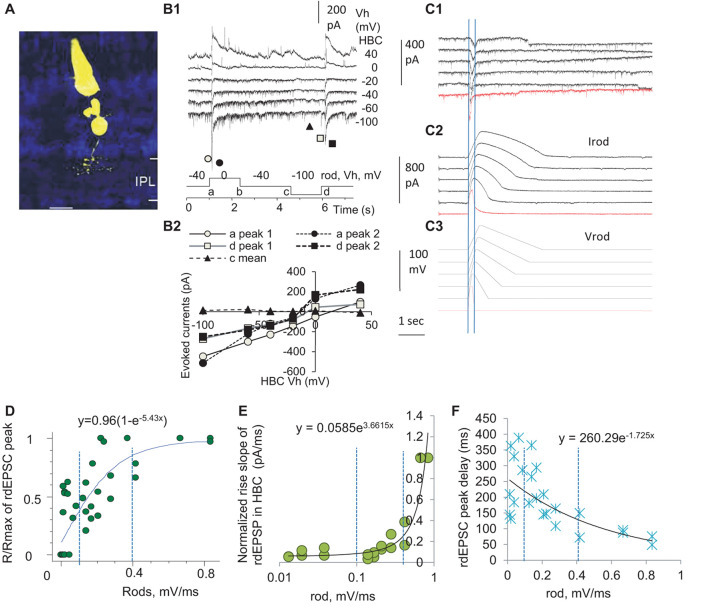
Three zones of presynaptic depolarizing speed differentially determine the delay, duration, and amplitude of the output in the rod-HBC synapse. Rod-HBC pairs **(A)** were recorded under voltage-clamp mode in dark-adapted conditions, and Vh was −60 mV for HBCs and −40 mV for rods. **(A)** Recorded cells were visualized by Lucifer yellow fluorescence with a confocal microscope. **(B1)** Rod-driven HBCs show robust spontaneous postsynaptic currents (sPSCs). The HBC responds to the maximum depolarization speed (square waves) with large transient inward cation currents (components a and d) and the maximum hyperpolarization speed with very small outward cation currents (components b and c), demonstrating that the synapse largely favors presynaptic depolarization. sPSCs and the PSCs evoked by stimulating rods reverse near 0 mV **(B2)**, indicating that the photoreceptor input mediates cation currents in the HBC and the recording is less affected by inhibitory neurons. **(C)** rods were stimulated by the depolarizing voltage ramps (−40 to 20 mV, V_rod_) of variable rising and decay slopes, and currents in the rod (I_rod_) and rod-driven excitatory postsynaptic currents (rdEPSCs, **C1**) in the HBC are recorded. Two vertical lines in **(C1)** denote the time to peak of rdEPSCs, which is much shorter and left-shifted compared with that of Vrod and Irod. **(D–F)** The normalized peak (the response/maximum response, R/R_max_, **D**), rising slope **(E)**, and delay **(F)** of the rdEPSCs were plotted as the function of the rod depolarization speed (mV/ms). **(D)** The amplitude of rdEPSC is exponentially correlated with the rod depolarization speed (*p* < 0.001) and plateaued at ∼0.4 mV/ms. **(E,F)** For the steeper rod depolarization, rdEPSCs rise faster **(E)** with a shorter delay **(F)**. The rising speed and peak delay of rdEPSCs are positively exponentially correlated with the rod depolarizing speed (both *p* < 0.001), and the turning point for the curve in **(E)** is ∼0.4 mV/ms. Two vertical dashed lines are placed at 0.1 and 0.4 mV/ms **(D–F)**.

**FIGURE 4:**
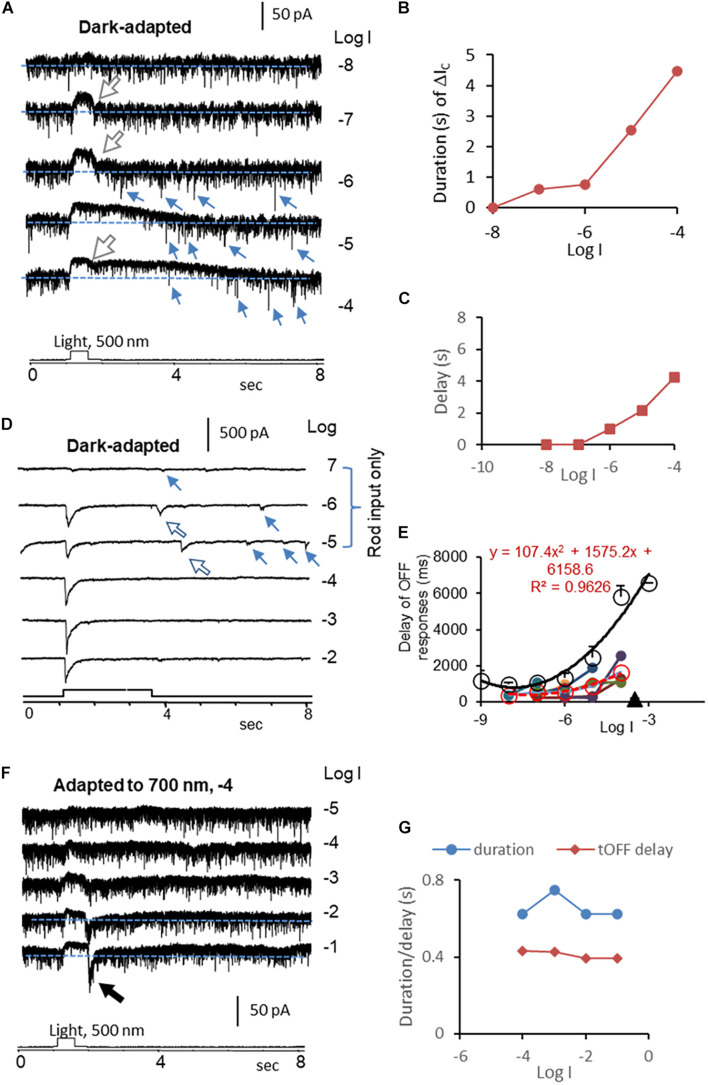
Kinetics of rod-driven OFF responses in HBCs and RGCs. **(A)** A rod-driven HBC was recorded for light-evoked cation currents (ΔI_C_, Vh = –60 mV) at different light intensities (log I) in dark-adapted conditions. The cell shows a high frequency of the transient excitatory currents (TECs), some of which appear after light offset and display an amplitude ≥ the absolute value of ΔI_C_ at the light onset, namely gigantic (G) TEC_offset_ (small arrow). ΔI_C_ is the outward sustained current, and the falling limb at the light offset (large white arrow) shows a short and stable delay. **(B,C)** The duration **(B)** of the ΔI_C_ in HBCs elongates upon increasing the light intensity like the light response of rods, and the first GTEC_offset_ **(C)** delays progressively longer upon increasing the light intensity, resembling that of the rod Phase 3 (Figure 2E). **(D)** ΔI_C_ (Vh = –60 mV) in a dark-adapted ON-OFF RGC shows OFF response only in the scotopic range (large arrow). The major transient ΔI_C_ at the light offset (ΔI_C__–__offset_, large arrow) is followed by a few minor ΔI_C__–__offset_s (small arrow). The latter appears at locations comparable with that of GTEC_offset_s in HBCs (A) and that of Phase 3 (Figure 2E) and action potentials (Figure 1E) in rods. **(E)** The delay time of the rod-driven OFF response varies among individual RGCs (color dot), and the mean value (red circle and dashed line) follows the trend of that of the rod Phase 3 (black circle and line) but generally faster. This supports that the scotopic ΔI_C__–__offset_ of RGCs is primarily mediated by rod-HBC synapses, and GTEC_offset_s of HBCs and the rod Phase 3 and action potentials play important roles. **(F)** The HBC in A shows transient (t) OFF response after being adapted to dim red light, when the duration of the entire ΔI_C_ and the delay of the ΔI_C_ at the light offset resemble that of cones **(G)**. The intensity of unattenuated [0 in log unit (log I)] 500 nm light from a halogen light source was 4.4 × 10^5^ photons.μm^–2^.s^–1^.

To access the input-output relationship in the rod-HBC synapse, we performed the dual-electrode whole-cell patch-clamp recording on rod-HBC pairs (Figure 3). Rod-driven HBCs were identified by the large response to the stimulation of individual presynaptic rods (Figure 3B), axonal ramification level (Figure 3A), and/or the characteristic light-evoked cation currents (ΔI_C_) (Figure 4A) with a waveform quite similar to that of the dark-adapted rods and the robust large transient excitatory currents (LTECs) (Pang et al., 2004, 2008). Based on the values of rod signals obtained from the last section, we designed stimuli that mimicked the light response of rods. We used them and classic square waves to stimulate rods and observed responses of HBCs.

We first used classic square waves to simulate rods (Figure 3B), and we observed that the HBCs responded to the fastest rod depolarization (the rising limb of the square wave) with large transient postsynaptic currents (PSCs) (a conductance increase) and to the fastest rod hyperpolarization (the falling limb of the square wave) with very small PSCs (a conductance decrease), which reversed at −6.1 ± 3.7 mV. PSCs evoked by the depolarizing limb of square waves displayed two peak components, which were delayed 9.0 ± 1.3 ms and 28.6 ± 2.4 ms (*n* = 12), respectively, with the reversal potential slightly varied. Rod-driven HBCs did not respond to the rising limb of a hyperpolarizing square wave in rods shorter than 40 ms. The data indicate that rod inputs mediate transient cation currents in HBCs, and the asymmetric response of the rod-HBC synapse to the rod depolarization and hyperpolarization makes the synapse primarily responsive to the reduction of light intensity.

The decay time of PSCs in the HBCs was best fit to a standard exponential function with a τ between 38 and 851 ms (*n* = 12) at holding potentials (Vh) of −100 to 40 mV, and the fastest decay was observed at −40 to −60 mV. These data indicate that when the membrane potential of the HBC is between −40 to −60 mV, the rod-HBC synapse has the best temporal resolution. The delay of PSCs was not significantly affected by the membrane potential of HBCs. The evoked PSCs in HBCs that were recorded at the chloride equilibrium potential (−60 mV) were inward currents, namely rdEPSCs. The decay constant of the fastest rdEPSC was 46.6 ± 2.6 ms (*n* = 9) at −40 mV. Since rods may receive fast signals at light offset from coupled cones, the time limitation (40 ms + 46.6 ms) predicts the best resolution for cone-mediated off responses at rod-HBC synapses to be 11.5 Hz.

Since the darkening-induced membrane depolarization of rods under physiological conditions (Phase 3, Figures 1B,E) is much slower than the rising limb of square waves (Figure 3B), we created electric stimuli to simulate light responses of rods, which contained a Phase-1-like component, a fast-hyperpolarizing nose of 32 ms, a Phase-2-like component, and/or a Phase-3-like component of various slopes (Figure 3C), to simulate rods and observe the response of HBCs. The Phase-3-like component rather than the nose and Phase-2-like section evoked rdEPSC in HBCs, and the amplitude, rising slope, and delay of rdEPSCs were differentially exponentially correlated with the presynaptic depolarization speed (Speed) (all *p* < 0.001) (Figures 3D–F). The response (R)- Speed curve plateaued at ∼0.4 mV/ms (Figure 3D), and the Rising slope—Speed curve turned at ∼0.4 mV/ms (Figure 3E). The Speed divided the curves into three different zones: the first zone of < 0.1 mV/ms corresponded to the rod native analog signal where the normalized amplitude of rdEPSCs (R/R_max_) grew with the increase of the Speed but the delay and duration were reduced weakly; the second zone of 0.1–0.4 mV/ms was in the range of analog coupled-cone signals where R/Rmax, rising slope, and delay time of rdEPSCs were all dramatically affected by the Speed; the third zone of > 0.4 mV/ms, which appeared to be a digital zone where R/Rmax of rdEPSCs reached the plateau and the duration and delay reached the *minimum*, resembling digital signals. In addition, compared to the peak of V_rod_ and I_rod_, the peak of rdEPSCs were dramatically left-shifted (Figure 3C). The stimulus-dependent shortening of rdEPSCs in HBCs is in contrast with the stimulus-dependent widening of light response in rods, while the latter is a typical analog signal, and the former is not.

Brief depolarization applied on rods that simulated rod APs could evoke the digital-like rdEPSC in HBCs (Figure 3C, red trace), consistent with previous studies from our laboratory (Pang et al., 2012b) and others’ (Cadetti et al., 2006), indicating that rod APs, as digital inputs, are transformed into digital-like output in the rod-HBC synapse.

The data together demonstrate that the rod-HBC chemical synapse can transfer both digital and analog signals to code for fast and slow changes of rod membrane potential. Rod-cone coupling is enhanced in light in this species, and the second Speed zone above, thus, involves rod signals coupled from cones when light intensity is above the cone threshold (Wu, 1988; Yang and Wu, 1989; Pang et al., 2012b), while the first and third Speed zones both involve scotopic OFF signaling.

### Rod-Driven OFF Responses in Hyperpolarizing Bipolar Cells and Retinal Ganglion Cells

**FIGURE 5:**
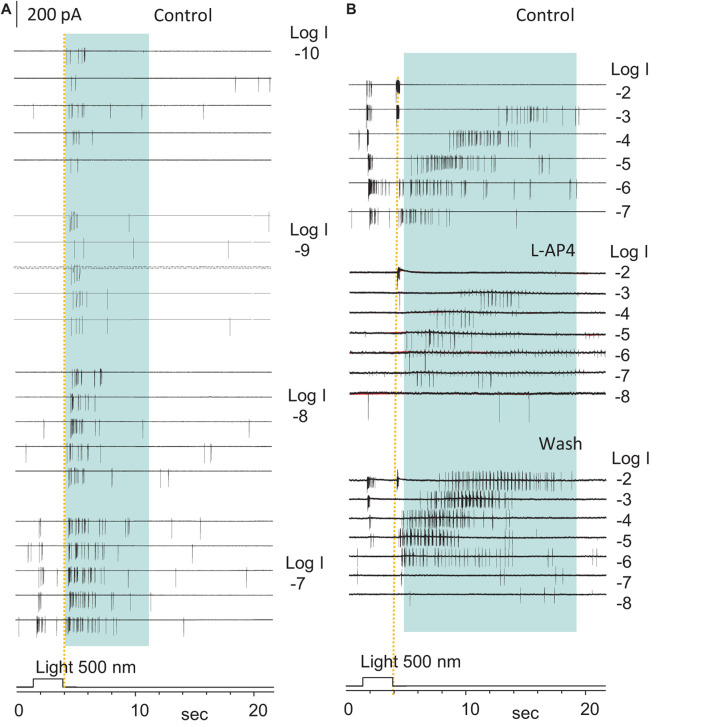
The highly sensitive excitatory rod-driven OFF response in dark-adapted RGCs. **(A,B)** Action potentials evoked by 500 nm light of a series of intensities (log I) in an ON-OFF retinal ganglion cell (RGCs) under the loose-patch mode. Action potentials are generated at both the light onset and offset, and the cell is identified as an ON-OFF cell. Light offset (yellow dashed line) evokes two spiking patterns, one with the short firing time, low light sensitivity, high firing rate, and short delay (cone-driven), and the other with a long firing period, very high light sensitivity, lower firing rate, and progressively longer delay for brighter light (rod-driven, in blue shaded area in **A,B**). The rod-driven OFF responses evoked by dim light (–10 to –6, **A,B**) showed a short delay, which is consistent with the delay of the rod Phase 3 at these light intensities in Figure 2E. The spikes at light onset is 3-log-unit less sensitive than those at light offset, which are completely and reversibly blocked by 5μM L-AP4 **(B)**. Spikes after light offset were less robust but maintained a similar pattern in L-AP4, indicating that that the rod-HBC synapse could mediate the highly sensitive excitatory response to light offset. The intensity of unattenuated [0 in log unit (log I)] 500 nm light from a halogen light source was 4.4 × 10^5^ photons.μm^–2^.s^–1^.

To determine the rod-driven OFF responses in RGCs, we first recorded the light-evoked spikes and cation currents in RGCs at different light intensities. The light-evoked excitatory cation current (ΔI_C_) in RGCs is primarily mediated by BCs (Pang et al., 2002a, 2003), and ΔI_C_ in BCs is mainly mediated by photoreceptors (Figure 3B; Wassle et al., 2009; Dowling, 2012; Masland, 2012). Thus, based on the light sensitivity and dynamic range of rods and cones (Yang and Wu, 1996), we identified the pure rod-driven transient OFF responses in RGCs (Figures4D,E, 5) at the light offset. In Figure 5, the ON-OFF RGC responded to the dim light of intensities between −10 and −8 log I with the rod-driven OFF response *without* ON response, and the delay of the first spike was comparable to that of the transient cone-driven OFF response at −3 and −2 log I. The ON response appeared around −7 log I. L-AP4 fully blocked ON responses, while both the rod- and cone-driven OFF responses became less robust but maintained the same pattern.

**FIGURE 6:**
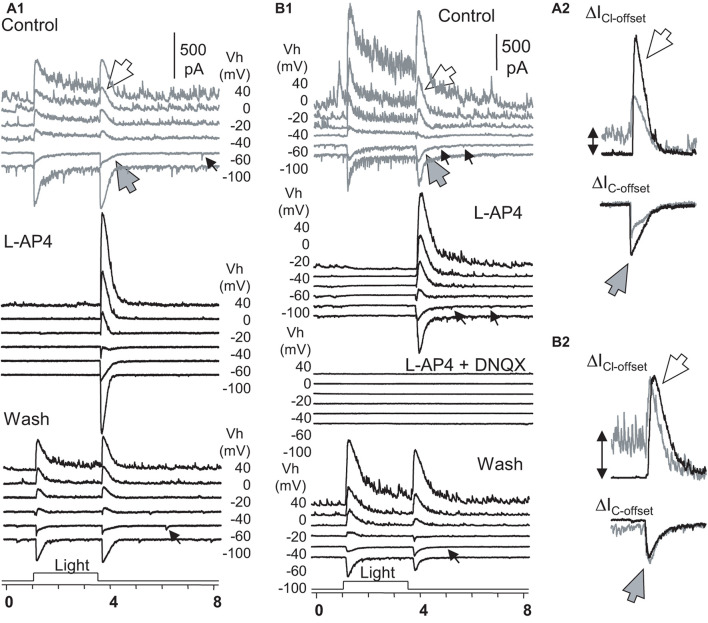
The role of ON and OFF pathways in the OFF response of RGCs. **(A,B)** Under Voltage-clamp conditions, ON-OFF RGCs were recorded for light-evoked currents at a series of holding potentials (Vh). L-AP fully reversibly blocked the light-onset-evoked transient and sustained currents **(A,B)** but did not affect or even enhanced the chloride (ΔI_Cl__–__offset_, large white arrow) and cation (ΔI_C__–__offset_, large gray arrow) currents at light offset **(A_2_,B_2_)**. In L-AP4, sustained currents during light ON are neglectable, indicating that they are primarily mediated by DBCs. **(A_2_,B_2_)** The L-AP4-induced increase of ΔI_Cl__–__offset_ cannot be fully accounted for by the loss of the sustained ΔI_C_ component during the light ON. The minor ΔI_C__–__offset_s (small arrow) that resemble the rod-driven component are blocked in A_2_ but not in B_2_. All L-AP4-resistant activities are fully reversibly blocked by DNQX **(B)**. The results, combined with data in Figure 5, indicate that RGC OFF responses are primarily mediated by DNQX-sensitive synapses, and the ON pathway could preferentially suppress inputs of OFF amacrine cells to RGCs, enhancing excitatory OFF response in RGCs. RGC, retinal ganglion cell; HBC, hyperpolarizing bipolar cell; DBC, depolarizing bipolar cell. The intensity of unattenuated [0 in log unit (log I)] 500 nm light from a halogen light source was 4.4 × 10^5^ photons.μm^–2^.s^–1^.

To better understand the role of the ON and OFF pathways in the OFF response, we examined ON-OFF RGCs (*n* = 17) for the effect of L-AP4 or CPPG (*n* = 3) on the light-evoked cation (ΔI_C_) and chloride (ΔI_Cl_) currents in the voltage-clamp condition (Figure 6). The two drugs fully and reversibly blocked responses of RGCs to light onset (ΔI_C__–__onset_ and ΔI_Cl__–__onset_), but the responses to light offset (ΔI_C__–__offset_ and ΔI_Cl__–__offset_) were unaffected or enhanced. DNQX reversibly blocked the OFF responses. Thus, in our experimental conditions, the OFF responses were mediated by DNQX-sensitive synapses, and all the ON responses were L-AP4 or CPPG-sensitive. L-AP4 and CPPC enhanced the amplitude of I_C__l__–__offset_ and I_C__–__offset_ averagely by 40% and 75%, respectively. The effect was more dramatic for I_Cl__–__offset_ (*n* = 9) or I_C__–__offset_ (*n* = 8) in different cells. Since L-AP4 enhanced ΔI_C__l__–__offset_ more dramatically than ΔI_C__–__o__nset_ for some RGCs, we deduced that the ON pathway could enhance OFF response by inhibiting OFF amacrine cells, accounting for the L-AP4-induced reduction of the extracellularly recorded firing rate after light offset in Figure 5B.

In 115 RGCs that we examined, 8% of RGCs displayed the pure rod-driven transient ΔI_C_ at the light offset (ΔI_C__–__offset_) (Figures 4D,E). The data demonstrate that rods can independently and predominantly mediate the scotopic transient OFF signals in RGCs. The native rod signals were temporally accurate to report the light offset to RGCs at low light intensities (≤−6 log I) but less accurate at the mesopic and photopic range (>−6 log I).

We then quantitively analyzed the delay of ΔI_C__–__onset_ and ΔI_C__–__offset_ in RGCs and compared them with that of the light response of rods, cones, and HBCs. In dark-adapted conditions, rod-driven HBCs were characterized by the high frequency of the transient excitatory currents (Pang et al., 2004, 2008) (TECs) which were robust in darkness and inhibited by light. Some TECs appeared after light offset with an amplitude ≥ the absolute value of ΔI_C__–__onset_, namely gigantic (G)TEC-_offset_ (Figure 4A). Light evoked primarily the sustained outward ΔI_C_ in the HBCs, and the light sensitivity and waveform resembled the photocurrents of rods (Figure 1B). Upon increasing the light intensity, ΔI_C__–__offset_ (the dip at the light offset) became shallower, the entire duration of ΔI_C_ elongated (Figure 4B), and the first GTEC_offset_ delayed longer (Figure 4C). The delay of the first GTEC_offset_ (Figure 4C) was comparable with that of Phase 3 (Figure 2E) and action potentials (Figure 1E) of rods but shorter. The signals were speeded up in the rod-HBC synapse, consistent with the data in Figure 3C. These results support that rods mediate the scotopic ΔI_C_ in rod-driven HBCs, and GTEC_offset_s are closely related to action potentials of rods.

ΔI_C__–__offset_ was delayed variably among RGCs, and it was often longer than ΔI_C__–__onset_. In dark-adapted retinas, ΔI_C__–__offset_ in some RGCs (*n* = 6 cells) showed a progressively dramatically elongated latency (averaged 365–1629 ms) upon increasing light intensity from −8 to −4, which (Figures 4D,E) followed the trend of that of the rod Phase 3 (Figure 2E) but shorter. The delays of the two datasets were well fit to similar polymodal functions (*p* = 0.003 and *p* = 0.0002, respectively), indicating that these ΔI_C__–__offset_s of RGCs are initiated in rods. The longest latency of the rod-driven ΔI_C__–__offset_ in these RGCs (ranged 1089–2541 ms) was about 1/3 of that of the rod Phase 3 at −4 log I and 6–10 times longer than that of the cone Phase 3 at −4 and −3 log I (168.5 ± 13.9 ms, *n* = 31) (Figure 2G). The improved kinetics in RGC ΔI_C__–__offset_s can be accounted for by the acceleration mechanism of the rod-HBC synapse revealed in the above section (Figures 2C–F). Following the major rod-driven ΔI_C__–__offset_, there were also several smaller transient inward currents (minor ΔI_C__–__offset_), whose temporal distribution and light sensitivity were comparable to that of GTEC_offset_s in HBCs (Figure 4A) and that of rod action potentials (Figure 1E). These results together indicate that the scotopic excitatory OFF response in some RGCs is purely mediated by the rod-HBC synapse and involves both analog and digital inputs from rods. The temporal resolution of rod signals was improved in rod-HBC synapses and HBC-RGC synapses. Current data further indicate that the native analog and digital rod inputs mediate the excitatory scotopic ΔI_C__–__offset_ (analog and digital-like) and GTEC_offset_s (digital-like), respectively, in HBCs for signaling the offset of scotopic light. Coupled cones in this species could mediate the graded and digital-like transient ΔI_C__–__offset_ in rod-driven HBCs, while the rod-cone coupling is enhanced by light (Wu and Yang, 1988; Yang and Wu, 1989). In the dim-light-adapted rod-driven HBC, light offset evoked transient excitatory ΔI_C__–__offset_ (Figures 4F,G) with the kinetics resembling that of cones, consistent with the notion that the rod-HBC synapse also mediates mesopic and photopic cone signals in the same HBCs and RGCs.

ΔI_C__–__onset_s of RGCs driven by rods and cones showed a well-integrated peak in dark-adapted conditions (Figure 4D), consistent with the similar kinetics found in Phase 1 of rods and cones (Figure 2). However, ΔI_C__–__offset_ of some ON-OFF RGCs was skipped or very small at −4 and/or −3 log I, at least in 7.25 s after the light offset, which is interpretable by the temporal separation of Phase 3 of rods and cones due to the differential delay time in addition to the distinctive light threshold of rods and cones.

## Discussion

### Rod-Hyperpolarizing Bipolar Cell Synapses Use Digital and Analog Inputs and Outputs

The rod-HBC synapse is known as a graded potential synapse, however, the stimulus-dependent shortening of PSCs in HBCs is in contrast with the light-dependent widening of light response of rods and HBCs. Rods use ribbon and non-ribbon (Chen et al., 2013) synapses and have been accepted to report light signals only by the graded membrane *hyperpolarization* (Matthews and Fuchs, 2010; Sanes and Zipursky, 2010). However, rods in the human retina also generate the Na^+^-dependent action potential (Kawai et al., 2001; Kawai et al., 2005) upon depolarizing to ≥ −50 mV. Ca^2+^ spikes have also been reported in the toad retina (Fain et al., 1980), as well as in the tiger salamander retina upon turning off bright light (10^3^ photons μm^–2^s^–1^) (Xu et al., 2005). It has been unknown whether and how a neuronal synapse in the central nervous system, including ribbon synapse, transmits both graded potentials and action potentials. Rod action potentials once were thought to selectively amplify the OFF response (Kawai et al., 2001, 2005) or generate negative afterimages (Xu et al., 2005) because of the long delay, but the synaptic mechanism for both the graded and action potential of rods to mediate the scotopic OFF response has been missing.

This study shows for the first time that the rod-HBC synapse can encode the speed of the rod *depolarization to* process both the analog and digital signals. We showed that dark-adapted rods responded to the light offset with both the action potential and graded depolarization, which had a shorter latency for dimmer light stimuli. With the speed coding mechanism, the analog input below 0.4 mV/ms, including the native scotopic rOFF and the mesopic cone-driven OFF inputs, evokes smaller rdEPSCs/the analog output. This appears to courage the signal integration in HBCs for better light sensitivity. Digital or digital-like inputs above 0.4 mV/ms, including rod action potentials (∼4 mV/ms) and photopic OFF signals from coupled cones, evoke the saturate rdEPSC / the digital-like output of maximum amplitude and minimum duration and delay. This appears to discourage the signal integration in HBCs and enhance the temporal and spatial resolution. This coding mechanism may also prevent the HBCs from being overexcited by strong photopic signals, serving as a protective gate. Such a unique “anadigital” synapse appears to be very beneficial for animals.

The “speed-coding” probably involves multiple factors. First, the fast membrane depolarization facilitates the synchronized multi-vesicle release at ribbon synapses in rod-driven HBCs (Pang et al., 2008). Rod-driven HBCs receive 75% of their inputs from rods and 25% from cones (Pang et al., 2004), and 80% of HBC dendritic contacts with photoreceptors in the salamander retina are invaginating ribbon junctions (Lasansky, 1978). The spontaneous and evoked neurotransmitter releases may use distinctive mechanisms (Cork et al., 2016; Kavalali, 2015). Rod can release glutamate via the fairly fast, ribbon, and nano-domain exocytosis (Jarsky et al., 2010). The fast rod action potential depolarization likely recruits the fast release processes. The docked vesicles and Ca^2+^ channels at the active zone of ribbons exhibit variable distances (Beaumont et al., 2005), and the rod action potential and the membrane depolarization > 0.4 mV/ms is likely to trigger the near-simultaneous Ca^2+^ channel opening at multiple ribbon bases to synchronize the synaptic vesicle exocytosis, saturating the amplitude and mostly shortening the duration of rdEPSCs. Second, the non-ribbon exocytosis is regulated by the calcium-induced calcium release (Cadetti et al., 2006; Babai et al., 2010; Chen et al., 2014). Although it likely primarily underlies the glutamate release in darkness, sustained rod depolarization over 200 ms has been shown to enhance glutamate release via Ca^2+^-induced Ca^2+^ release (Cadetti et al., 2006; Babai et al., 2010; Chen et al., 2014). The rod action potential and depolarization longer than 200 ms and faster than > 0.4 mV/ms probably use both mechanisms to mediate the digital-like rdEPSC and GTEC_offset_s in HBCs, underlying the excitatory ΔI_C__–__offset_ in RGCs. Besides, glutamate transporters remove glutamate from the synaptic cleft with a cycle time and plateau speed (Lupfert et al., 2001; Mim et al., 2005; Akyuz et al., 2013), and the synchronized glutamate release in the rod-HBC synapse prevents iGluRs from desensitization (Pang et al., 2012b). These factors also likely contribute to the speed-coding phenomenon in the rod-HBC synapse. In an earlier study on the calcium-dependent neurotransmitter exocytosis in Mb1 BCs from the goldfish retina (von Gersdorff and Matthews, 1994), two components of membrane retrieval with distinctive kinetics were observed following secretory stimulation, suggesting that the speed coding strategy is probably not restricted to the rod-HBC synapse in the retina.

Graded-potential neurons should not fire action potentials, while recent data appear to violate this general rule (Fain et al., 1980; Protti et al., 2000; Kawai et al., 2001, 2005; Saszik and DeVries, 2012). Some of the findings were obtained from diseased retinas (Kawai et al., 2001, 2005). We observed light-evoked action potentials in outer retinal neurons under normal membrane potential levels and was usually larger at the beginning of the patch recording, which indicates that these action potentials are physiological.

The delayed OFF response mediated by action potentials in rods has been previously reported in some horizontal cells (Xu et al., 2005). In our results, dark-adapted RGCs showed pure rOFF responses followed by a few minor OFF responses with variable delay times, whose temporal distribution was in line with that of GTEC_offset_s accountable by rod action potentials and rod Phase 3. The response of the rod-driven HBC to rod action potentials appears to be different from that of horizontal cells (HCs), which is likely because of the difference in their synaptic structure (Pang et al., 2008), connection (Mariani, 1984; Zhang J. et al., 2006; Wu, 2010), the subtype of iGluRs (Yang et al., 1988; Pang et al., 2008), and the extent of signal convergence (Zhang A. J. et al., 2006; Zhang A. J. and Wu, 2009). In the dark-adapted salamander retinas, TECs were found only in rod-driven HBCs (Wu et al., 2000; Pang et al., 2004, 2008) but not HCs (Yang et al., 1988; Xu et al., 2005), and similar miniature currents were reported in mammalian AII amacrine cells postsynaptic to rod BCs (Pang et al., 2007). Previous works have found that brief depolarization could modify the glutamate release at the rod-horizontal synapse (Cadetti et al., 2005; Chen et al., 2013), while this study tested the effect of both rod depolarization and hyperpolarization on HBCs. The rod light response is primarily hyperpolarizing and that evoked by brighter light stimuli shows a brief hyperpolarizing nose in Phase 1 (traces at ≥ −4 in Figure 1E; Pang et al., 2012b), and our results demonstrate that the trailing edge of a membrane hyperpolarization with a duration < 40 ms could not evoke OFF responses from HBCs.

### Rod-Hyperpolarizing Bipolar Cell Synapses Can Signal the Darkening in the Vertebrate Retina With or Without Cones’ Assistance

How rod-HBC synapses work for OFF pathways has been unclear because rods do not immediately depolarize at light offset (Toyoda et al., 1970; Xu et al., 2005; Pang et al., 2012b; Fortenbach et al., 2015). Results from this study showed that rod-HBC synapses could signal the darkening of light in the vertebrate retina via the light offset-induced (1) graded depolarization and (2) firing of action potentials of rods, (3) signals from coupled cones, (4) GTEC_offset_ in HBCs accountable by rod action potentials, and (5) graded rdEPSCs, and (6) digital-like rdEPSCs in HBCs. We also observed scotopic OFF responses in RGCs that were temporally accurate and resistant to L-AP4. Given that L-AP4 did not suppress I_C__–__offset_s in all RGCs tested, the minor I_C__–__offset_s in some RGCs were blocked by DNQX but not L-AP4, and cones cannot be activated by scotopic light, these data further indicate that rod-HBC synapses or iGluRs can mediate the excitatory *scotopic* OFF response at light offset without cones’ assistance.

In ON-OFF RGCs, the OFF response could be absent in some light intensity (previously termed the “dip”) (Olsen et al., 1986; Hensley et al., 1993), and the mechanism has been unclear. The Hill equation could well predict rod-cone-driven ON responses (Hensley et al., 1993; Pang et al., 2016) but not rod-cone-driven OFF responses. Our results revealed that ΔI_C__–__onset_s of RGCs driven by rods and cones showed a well-integrated peak in dark-adapted conditions consistent with the similar kinetics found in Phase 1 of rods and cones. ΔI_C__–__offset_ of RGCs was absent or very small at −4 and/or −3 log I, at least in the 7.25 s after the light offset, interpretable by the temporal separation of Phase 3 of rods and cones (> 600 ms) due to the different latency in addition to the distinctive light threshold. Because of the lower light sensitivity of cones compared to that of rods in both the salamander and human retina, the rod-cone coupling (the secondary rod pathway for mammals) is not able to mediate *scotopic* OFF responses in RGCs.

RGCs may generate “transient” and “sustained” OFF responses. Although “OFF responses” appear to involve OFF pathways, the former is the membrane *depolarization at the light offset* while the latter is the membrane *hyperpolarization at the light onset*. Previous and current data have shown that HBCs respond strongly to the fastest depolarization of rods (Rabl et al., 2006; Li et al., 2010), and our study also revealed that rod-driven HBCs only weakly responded to the hyperpolarization of rods. We revealed an asymmetry for the response of the rod-HBC synapse to the rod depolarization and hyperpolarization. It, consistent with previous observations in RGCs (Pang et al., 2003; Arman and Sampath, 2012), demonstrates that the rod-HBC synapse, the mammalian tertiary rod pathway, is primarily responsive to the reduction of light intensity and mediate excitatory transient OFF responses in RGCs. Furthermore, we reported that the native rod input contributed to the transient scotopic excitatory ΔI_C__–__offset_ and GTEC_offset_ in rod-driven HBCs for signaling the offset of scotopic light, and the rod input from coupled cones could mediate the excitatory ΔI_C__–__offset_ and photopic OFF signals. ON pathways likely mediate the sustained OFF response in RGCs (Pang et al., 2003; Arman and Sampath, 2012).

Moreover, L-AP4 could reduce extracellularly recorded spikes evoked by the light offset in some RGCs in our results, comparable to a previous finding in OFF RGCs (Protti et al., 2005). Meanwhile, our data also showed that in half RGCs, L-AP4 enhanced ΔI_Cl__–__offset_s more than ΔI_C__–__offset_s. Therefore, it is likely that the ON pathway could inhibit OFF amacrine cells (ACs) to influence the excitatory OFF response in RGCs. Such influence probably involves glycinergic ACs (Protti et al., 2005) and could be more important when the membrane potential is depolarized above the chloride equilibrium potential. Besides, we did not see action potentials in cones in salamander retinas. It is unclear whether this is due to the membrane potential or/and calcium signaling. Salamander cones express the calcium-binding protein calbindin D-28k (Zhang J. and Wu, 2009), while rods do not. Due to the lower light sensitivity of cones, dimer light hyperpolarizes rods more profoundly than cones. Cones can generate fast responses to light offset, whose kinetics and light sensitivity are largely different from the scotopic OFF response in HBCs and RGCs.

### Rod-Hyperpolarizing Bipolar Cell Synapses Mediate the Rod-Driven OFF Response in Retinal Ganglion Cells and Improve the Temporal Resolution of the Signals

Our data revealed the excitatory transient scotopic OFF responses in RGCs, including action potentials and ΔI_C__–__offset_. These OFF responses exhibited a progressively longer delay upon increasing light intensity and followed the trend of the rod Phase 3, indicating that they are driven by rods and rod-HBC synapses. Meanwhile, the scotopic ΔI_C__–__offset_ in RGCs was composed of several small peaks with the temporal distribution like that of GTEC_offset_s in the HBCs and the action potential and Phase 3 of rods, supporting the involvement of digital inputs. Light enhances the rod-coupling in the salamander retina (Wu and Yang, 1988; Yang and Wu, 1989). In our experimental conditions, rod-driven HBCs generated transient OFF responses near the cone Phase 3 in the dim-light-adapted retinas, consistent with the notion that coupled cones feed fast inputs to the rod-HBC synapse and can mediate mesopic and photopic OFF responses in the same HBCs and RGCs.

The delay of the rOFF response in RGCs was shorter than that of the rod Phase 3 and the ΔI_C__–__offset_ and first GTEC_offset_s of HBCs, which can be explained by the accelerating effect of the rod-HBC synapse. Our data showed that the rod-HBC synapse left-shifted the peak of signals and shortened the duration, which could enhance the frequency responsiveness/temporal contrast of visual signals passing the synapse. The best temporal resolution of the scotopic OFF response in RGCs (e.g., a delay of 365 ms corresponding to 2.74 Hz) in our results is aligned with the bandpass-filter property of rod-rod electric synapses and rod-BC synapses (Armstrong-Gold and Rieke, 2003; Zhang and Wu, 2005), while that of the photopic ones (e.g., 11.5 Hz calculated per the kinetics of rdEPSCs) in our results is aligned with the filter property of rod BCs reported previously (Cangiano et al., 2007). A mathematic model to transform rod signals into cation currents in RGCs is still absent.

## Materials and Methods

### Animals

Laval tiger salamanders (*Ambystoma tigrinum*) were purchased from Charles D. Sullivan, Co. (Nashville, TN, United States) and KON’s Scientific Co. Inc. (Germantown, WI, United States) and handled per policies on the treatment of laboratory animals of Baylor College of Medicine and the National Institutes of Health, including the housing, transportation, food, euthanasia, etc. Animals were dark-adapted for 1–2 h prior to the experiment. Before each experiment, salamanders were anesthetized in MS222 until the animal gave no visible response to touch or water vibration. The animals were then quickly decapitated, and the eyes were enucleated. The investigators understand the ethical principles under which the journal operates and that the work complies with the animal ethics checklist as described in the Editorial by Grundy (2015). Chemicals were purchased from Sigma-Aldrich (St. Louis, MO) and Tocris Bioscience (Bristol, United Kingdom) except otherwise specified.

### Dual-Cell Patch-Clamp Recording of Rod-Hyperpolarizing Bipolar Cell Pairs

All procedures were performed under infrared (∼1 mm) illumination with dual-unit Nitemare (BE Meyers, Redmond, WA) infrared scopes. The whole-cell patch-clamp recording (Pang et al., 2012a; Gao et al., 2013), preparation of living retinal slices (Werblin, 1978; Wu, 1987), light simulation, immunofluorescence, and confocal microscopy (Pang et al., 2008, 2012b) essentially followed procedures described in previous publications.

We recorded rod-BC pairs from retinal slices and rods and RGCs from slices and flat-mount retinas under the whole-cell voltage- and current-clamp conditions. BCs that did not respond to depolarizing rods were not included. We held the membrane potential of neurons to the chloride or cation equilibrium potential (E_Cl_ and E_C_, respectively) for separately studying the excitatory postsynaptic current (cation current, ΔI_C_, recorded at E_Cl_) mediated by rods or BCs and the inhibitory postsynaptic current (chloride current, ΔI_Cl_, recorded at E_C_) mediated by amacrine cells. An Axopatch 700B amplifier was connected to a DigiData 1322A interface and operated by the pClamp software v9.2 and v10.3 (Axon Instruments, Foster City, CA). Patch pipettes had 5–8 MΩ tip resistance when filled with an internal solution containing 112 mM Cs-methanesulfonate, 12 mM CsCl, 5 mM EGTA, 0.5 mM CaCl_2_, 4 mM ATP, 0.3 mM GTP, 10 mM Tris, and 0.5% Lucifer yellow, adjusted to pH 7.3 with CsOH. For current-clamp and some voltage-clamp recordings, the pipettes were filled with internal solutions containing: 112 mM K-gluconate, 10 mM KCl, 10 mM EGTA, 10 mM HEPES, 0.5 mM CaCl_2_, 1 mM MgCl_2_, 4 mM Na_2_-ATP, 0.3 mM Na_3_-GTP, and 0.5% Lucifer yellow, adjusted to pH 7.3 by KOH. The bath was maintained at room temperature (20–23 °C) and superfused continuously with oxygenated Ringer solution containing (in mM) 108 mM NaCl, 2.5 mM KCl, 1.2 mM MgCl2, 2 mM CaCl2, and 5 mM HEPES, adjusted to pH 7.3. All pharmacological agents were dissolved in Ringer solution and pH was re-adjusted. The internal solution and the external normal Ringer’s solution yield an E_Cl_ of −59 mV. Recorded cells were visualized by Lucifer yellow fluorescence with a confocal microscope LSM 510 and LSM 800, Carl Zeiss, Germany). L-AP4 was purchased from Tocris (0103, Bristol, United Kingdom) and applied in the bath. Other chemicals were purchased from Sigma-Aldrich (St. Louis, MO).

A photostimulator delivered light spots of a diameter of 600–1,200 μm and 500 nm wavelength (λ_max_ = 500 nm, full width-half max 10 nm) at a series of intensities (−10 to −1 log I) to stimulate the retina via the epi-illuminator of the microscope (Maple and Wu, 1998; Pang et al., 2002b, 2010a). Since we delivered uncollimated light beams through an objective lens of a large numerical aperture (Zeiss 40x/0.75 water), the incident light could enter the retina in many directions and, thus, had a minor photoreceptor self-screening effect (Field and Rieke, 2002). The intensity of unattenuated [0 in log unit (log I)] 500 nm light from a halogen light source was 4.4 × 10^5^ photons.μm^–2^.s^–1^.

### Statistics

Data were analyzed with Sigmaplot v11.0 (Systat, Point Richmond, CA), Clampfit v9.2 and v10.3 (Axon Instruments, Foster City, CA), and Microsoft Excel v1708 (Microsoft Co., Redmond, WA) software and presented by mean ± SEM Regression analysis and student’s *t-*test were performed, and the two-tailed *p*-value was reported in all cases. The peak amplitude, rising slope, and delay of responses (R) of HBCs were plotted against light intensity (log I) and the rod depolarizing speed, which were well fit by a standard exponential function $f\,\left(x\right)=\sum_{i=1}^{n}R_{i}\,e^{-x/\tau_{i}}$, a linear or an exponential cumulative distribution function $f\,\left(x\right)=\sum_{i=1}^{n}T_{i}\,\left(1-e^{-x/\tau_{i}}\right)$. The data collection was completed before data analysis and was independent of data interpretation. Studies on rod-HBC pairs excluded light responses of a decay or delay time longer than 6566 ms and HBCs that were not responsive to depolarizing rods. The α level for rejecting the null hypothesis was 0.05.

## Data Availability Statement

The original contributions presented in the study are included in the article/supplementary material, further inquiries can be directed to the corresponding author/s.

## Ethics Statement

The animal study was reviewed and approved by the Institutional Animal Care and Use Committee.

## Author Contributions

This work was conducted in the Department of Ophthalmology, Baylor College of Medicine. J-JP contributed to study design, data acquisition, data analysis and interpretation, and drafting and revising the manuscript. FG contributed to data acquisition and analysis. SW contributed to funding support, materials, and study design. All authors approved the final version of the manuscript and agreed to be accountable for all aspects of the work in ensuring that questions related to the accuracy or integrity of any part of the work were appropriately investigated and resolved and designated as authors qualify for authorship, and all those who qualify for authorship are listed.

## Conflict of Interest

The authors declare that the research was conducted in the absence of any commercial or financial relationships that could be construed as a potential conflict of interest.

## Publisher’s Note

All claims expressed in this article are solely those of the authors and do not necessarily represent those of their affiliated organizations, or those of the publisher, the editors and the reviewers. Any product that may be evaluated in this article, or claim that may be made by its manufacturer, is not guaranteed or endorsed by the publisher.

## Abbreviations

HBC: hyperpolarizing bipolar cell
RGC: retinal ganglion cell
Δ I_C_: light-evoked cation current
Δ I_Cl_: light-evoked chloride current
Log I: light intensity in log unit of the attenuation of the light
EPSC: excitatory postsynaptic current
rdEPSC: EPSC evoked by electrically stimulating rods
TEC: transient excitatory current in HBCs
GTEC_offset_: gigantic transient excitatory current after light offset in HBCs
Vh: holding potential.
